# 

**Published:** 1890-02

**Authors:** 


### CONTENTS.

### COMMUNICATIONS.

Dental Education—Office Pupilage....57
Evolution in Dentistry....61
Is the Six Year Molar less Perfect than the Other Teeth?....67
Functions of Tooth Pulp....69

### SELECTIONS.

The Anatomy of the Future....71
The Graphic Arts in Medicine....73
Virchow and the Darwinian Theory....74
Pure Air in Churches....74
Integrity in Medical Colleges....76
Digestibility of Boiled Milk....77
The Drink Question in France....78
Work Kills no Man....78
Antiseptic Properties of Corrosive Sublimate in Minimal Quantities....79
Hygiene and Sunday....81
Mouth Washes. Food and Sleep....82
Peroxide of Hydrogen as Antiseptic and Disinfectant....83
Thumb Sucking....84
Preventive Therapeutics. Mercuric Chloride. Sterilized Lint. Local Anæsthesia 85
Treatment of Hemorrhage after Extraction of Teeth. Diet of Strong Men....86
Gastric Digestion....87
Medical Congress....88
The Chinese Way. Headache....89
Wounds of Inferior Maxillary Bone....90
Operation and Prognosis of Lingual Cancer. A Belgian View of Medical Advancement....91
Intertrigo. Four at a Time....92
Decomposition of Iodoform Solutions. Bacillus of Tetanus....93
Miscellaneous Items....94, 95
Certain Effects of Tobacco Smoking. Colored Spectacles....96
Rules for Good Health. Eating Before Sleeping....97
The Practical Physician. Miscellaneous Items....98, 99
The Normal Diet. Number of Dentists in Germany....100
Transmission of Scarlatina. Treatment of Tetanus....101
Miscellaneous Items....102
Cocaine. Bacteriology....103
Treatment of a Cold. Association News....104

### EDITORIAL.

An International Dental Congress....105
Editorial Change....106
Professor C. L. Ford's Questions....107

